# Poly(Dimethylsiloxane) (PDMS) Affects Gene Expression in PC12 Cells Differentiating into Neuronal-Like Cells

**DOI:** 10.1371/journal.pone.0053107

**Published:** 2013-01-03

**Authors:** Joanna M. Łopacińska, Jenny Emnéus, Martin Dufva

**Affiliations:** Department of Micro- and Nanotechnology, Technical University of Denmark, Kongens Lyngby, Denmark; Northwestern University Feinberg School of Medicine, United States of America

## Abstract

**Introduction:**

Microfluidics systems usually consist of materials like PMMA - poly(methyl methacrylate) and PDMS - poly(dimethylsiloxane) and not polystyrene (PS), which is usually used for cell culture. Cellular and molecular responses in cells grown on PS are well characterized due to decades of accumulated research. In contrast, the experience base is limited for materials used in microfludics chip fabrication.

**Methods:**

The effect of different materials (PS, PMMA and perforated PMMA with a piece of PDMS underneath) on the growth and differentiation of PC12 (adrenal phaeochromocytoma) cells into neuronal-like cells was investigated using cell viability, cell cycle distribution, morphology, and gene expression analysis.

**Results/Conclusions:**

After differentiation, the morphology, viability and cell cycle distribution of PC12 cells grown on PS, PMMA with and without PDMS underneath was the same. By contrast, 41 genes showed different expression for PC12 cells differentiating on PMMA as compared to on PS. In contrast, 677 genes showed different expression on PMMA with PDMS underneath as compared with PC12 cells on PS. The differentially expressed genes are involved in neuronal cell development and function. However, there were also many markers for neuronal cell development and functions that were expressed similarly in cells differentiating on PS, PMMA and PMMA with PDMS underneath. In conclusion, it was shown that PMMA has a minor impact and PDMS a major impact on gene expression in PC12 cells.

## Introduction

Microfluidics provides the opportunity to investigate cells on both single and multi-cellular level with excellent spatial and temporal control of cell growth and stimuli [1]. Although microfluidics based cell culturing presents many advantages over conventional cell culturing methods, it is not yet widely used [2]. This may be due to that additional factors have to be considered before using microfluidics for biological experiments, e.g. the influence of flow conditions on the cells and the material used for system construction. While batch cultures are standardized using polystyrene (PS) flasks or microtitre plates, microfluidics devices are made of a whole range of other materials, such as poly(dimethylsiloxane) (PDMS), poly(methyl methacrylate) (PMMA), polycarbonate (PC), cyclic olefin copolymers (COC) and glass [3]–[6]. One reason for this is that PS is not straightforward to us for constructing microfluidics devices; the main challenge being to bond two pieces of PS together [4], [7].

Composite PDMS based devices, in which a PDMS layer is grafted onto another material like glass, PS, or PMMA, have become widely popular in the microfluidic field. The reason is that it is possible to create highly complex fluidic control features in PDMS, such as pumps and valves that control medium delivery to the cells [8]. We have recently developed a powerful way to create and drive microfluidic cell culturing systems using a modular approach, also containing PDMS parts [9], [10], based on a handful of components fabricated in PMMA and PDMS [11]–[14]. Although a significant number of PDMS-based microfluidic cell culture systems have been reported [5], [15]–[18], remarkably little attention has been paid to the specific properties of PDMS, which may potentially influence the biological results. Properties of interests are gas permeability, absorption of hydrophobic molecules and leaching of uncured oligomers from the polymer components into the cell culture medium [4], [19]. It has been reported that mouse mammary fibroblasts cultured in PDMS-based microchannels responded significantly different, when compared to culturing in a 96-well plates [20]. Furthermore, PDMS oligomers were detected in the plasma membranes of NMuMG cells cultured in PDMS microchannels for 24 hours [19]. Millet et al. [17] showed that the biocompatibility of PDMS microdevices may be significantly increased by several extractions/washes of PDMS with various solvents to remove impurities. Due to the extensive use of PDMS and its reported negative effects on cells, it is highly important to gather as much information as possible about its effects on cells in order to be able to predict the effect of PDMS on any given assay.

The aim of this study was to explore the biocompatibility of cell culturing on PMMA and PDMS in a configuration resembling our previously developed modular system [9], [10], [10,11], and compare it to cell culturing on PS as the reference material. The study also includes a model for composite PDMS chips where the control features are defined in PDMS while the cells are grown on glass, PS or PMMA [4].

Biocompatibility is often assessed using measurements of cell viability, growth, and morphology. However, these parameters are not sufficient to explain specific material effects on the molecular level [21] (Lopacinska, 2012). For instance, alterations in gene expression can underlie many diseases, e.g. neurodegenerative disorders such as Alzheimer’s disease [22]–[24]. Therefore, the cell experimental system must have a minimal impact, or at least a known impact, on the biological system since there is a link between gene expression and disease mechanism. The choice of investigated biocompatibility parameters is thus vital. For a general-purpose cell culture chip, a material is biocompatible when it: (i) supports high proliferation rates, (ii) does not induce cell death, and (iii) does not alter the transcriptome profile, compared to a reference material such as PS. In most microfluidics chips or even at the chip material level, especially the latter requirement is not well met or characterized. We therefore decided to analyze the gene expression profiles of cells by means of DNA microarray expression analysis to check if any differences between tested conditions can be observed. DNA microarrays are powerful tools that provide a means to correlate effects on gene expression profiles to fundamental biological processes [25], [26].

We previously reported that a PMMA based cell culture chip showed excellent biocompatibility using both viability assays and gene expression profiling of HeLa cells grown on PS, PMMA and inside PMMA chips [26]–[28]. The results strongly indicated that PMMA is a good candidate for fabrication of general-purpose cell culture chips, as PMMA had no effect even on Hela cells at the molecular level or cellular function level as compared to cells growing on PS. However, our previous studies on PMMA were limited to one cancer cell line (HeLa cells), which has been selected for growth, and only one biological condition - proliferation. Detailed investigation of the impact of cell culture chip-materials on other cell types and other cellular functions is highly needed to understand chip material-cell interactions.

The present study was therefore designed to explore the response of a more complex cellular model (PC12 cells) that included a detailed characterization of both growth and differentiation of these cells. The PC12 cell line represents a well-established *in vitro* model to examine neuronal cell differentiation, neurite outgrowth and neurosecretion [29], [30]. Upon nerve growth factor (NGF) treatment, PC12 cells stop dividing and start to extend neuritis and acquiring the phenotype of sympathetic ganglion neurons [31], [32]. Sympathetic-like neuron development is characterized by neurite outgrowth, electrical excitability, and the presence of synaptic vesicles [33]. This study answers question about the applicability of PMMA as a solid support material and *trans*-acting effects of PDMS on the complex rearrangement of biochemical constitution of dividing cells into non-dividing differentiated cells.

## Materials and Methods

### Cell Culture

Adrenal phaeochromocytoma cells (PC12), obtained from the German Collection of Microorganisms and Cell Cultures (The DSMZ), were cultured in laminin-coated 75 cm^2^ culture flasks (Easyflask, Nalgen Nunc International Rochester, USA) in D- MEM/F-12 (1∶1) with GlutaMAX™ (Invitrogen, USA) supplemented with 15% horse serum, 2.5% fetal bovine serum, 0.5% Hepes and 1% penicillin/streptomycin (all from Sigma, USA). The differentiation medium was composed of D-MEM/F-12 (1∶1) with GlutaMAX™ (Invitrogen, USA) supplemented with 0.5% horse serum, 0.5% fetal bovine serum, 0.2% of 0.05µg/µl nerve growth factor (NGF), 0.5% Hepes and 1% penicillin/streptomycin (all from Sigma, USA). Both growth and differentiation media were replaced every second day.

The cells were incubated at 37°C in a humidified atmosphere of 95% air and 5% CO_2_. PC12 cells were grown to 80–90% confluency and harvested with 0.1% trypsin, 0.02% EDTA in Ca^2+^ and Mg^2+^ -free phosphate-buffered saline (Sigma, USA). The cells, at passage number 8, were seeded into 3 different kinds of laminin-coated (10 µg/ml in PBS, Sigma, USA) cell culture dishes (100 mm×20 mm, Nalgene Nunc International Rochester, USA): (1) petri dishes (polystyrene – PS), (2) petri dishes with a 2 mm thick PMMA plate (Nordisk Plast, Denmark) placed at the bottom (PMMA), (3) petri dishes having a 2 mm thick PMMA plate with through holes, placed on top of a 2 mm thick PDMS layer (Sylgard 184, DowCorning) (PMMA-PDMS). PDMS was prepared by mixing a 10∶1 mass ratio of elastomer to curing agent and cured overnight at 65°C. All PMMA pieces were made by a CNC controlled micromilling machine (Folken, Glendale, CA). Tested materials were sterilized by using 0.5M NaOH. After 10 min of sterilization, NaOH was aspirated and samples were washed three times with a sterile 1xPBS. Two independent biological repeats for each culture conditions were prepared.

### Cell Viability and Metabolic Activity Assays

The calcein and propidium iodide assay was used to determine the number of live and dead cells on the investigated surfaces. Cells were seeded and cultured for 4 days at the concentration of 3×10^5^ cells per well in 6-well plates, containing the different test materials. After 4 days of cell culturing, the medium was removed and 2.5 ml of 3 µM calcein AM (staining live cells) and 3 µM propidium iodide (PI, staining dead cells) was added to each well. The dyes were incubated with cells for 30 min, after which the signal from calcein and PI were determined, using an automated inverted life science microscope (Axio Observer.Z1, Carl Zeiss). The experiments were repeated three times and for each experiment at least 100 cells were visualized and analyzed. The results were shown as the mean ± SD.

The MTT ((3-(4,5-Dimethylthiazol-2-yl)-2,5-diphenyltetrazolium bromide, a yellow tetrazole)) assay was utilized to evaluate PC12 cell growth on the test surfaces. Cells were cultured without NGF for 4 days, after which 2.5 ml of a 5 mg/ml solution of MTT in 1xPBS was added to each well. MTT was allowed to be metabolized by cells for 4 hours in 37°C and an atmosphere of 5% CO_2_. The medium was thereafter carefully removed from each well and the water insoluble product dissolved in 2.5 ml DMSO. The plates were shaken for 10 min and the optical density (OD) of the dissolved solute was read at 560 nm and the background at 670 nm subtracted. Each sample analysis was repeated three times and the results were reported as the mean ± SD.

### Cell Morphology Investigation

After 4 days of differentiation with NGF-treatment, the PC12 cells were fixed in 2% glutaraldehyde in 0.05M cacodylate buffer for 15–20 minutes at room temperature, and subsequently washed in 1xPBS. Cell morphology was investigated by using an automated inverted life science microscope (Axio Observer.Z1, Carl Zeiss) equipped with 40x magnification objective for phase contrast and PlasDIC. The number of differentiated cells was determined by manual counting of cells that had at least one neurite with a length equal to the cell body diameter. The data was presented as the percentage of the total number of cells for each experiment, with each experiment repeated three times, and where at least 100 cells were visualized and analyzed. The results were shown as the mean ± SD.

### Cell Cycle Analysis and Sub-G1 DNA Measurement

PC12 cell were cultured at the three different test conditions (PS, PMMA, PMMA-PDMS) with and without NGF treatment, and then analyzed by ﬂow cytometry assays. Brieﬂy, cells were harvested every 24 hours by centrifugation at 200×g for 10 min, washed with ice-cold PBS and fixed with 70% cold ethanol at 4°C overnight. The fixed cells were suspended in PBS, and further treated with RNase (DNase free, 170 µg/ml final concentration in PBS) and PI (45 µg/ml final concentration in PBS) for 30 min at 37°C in the dark. The cell suspension was stored at 4°C protected from light until analysis. The intensity of PI was measured using a Gallios Flow Cytometer (Beckman Coulter, USA). At least 10,000 cells were analyzed for each sample and gated on the basis of forward and side scatter. The number of cells in different phases of the cell cycle was analyzed using Cyflogic v. 1.2.1 and for DNA, doublets and higher order cell clumps were detected, collecting peak versus integrated signals. Error bars show mean ± SD estimated from results of three independent identical experiments.

### RNA Preparation

After 4 days of subculture, the non-differentiated PC12 cells were harvested from half of the seeded cell culture dishes and the total RNA was isolated and collected. The medium in the rest of the samples was changed to MEM/F-12 (1∶1) with GlutaMAX™ (Invitrogen, USA) supplemented with 0.5% horse serum, 0.5% fetal bovine serum, 0.2% 0.05µg/µl nerve growth factor, 0.5% Hepes and 1% penicillin/streptomycin (all from Sigma, USA). After 4 days differentiation the total RNA was isolated from differentiated PC12 cells grown on the various surfaces. The RNeasy total RNA isolation kit (Qiagen, USA) was used to isolate total RNA from the cells cultured under the different experimental conditions. Quantification of obtained total RNA was done using an Ultraspec 3000 spectrophotometer (Pharmacia Biotech, UK). To assess RNA quality, an Agilent 2100 Bioanalyzer (Agilent Technologies, USA) was utilized.

### DNA Microarray

**Figure 1:**
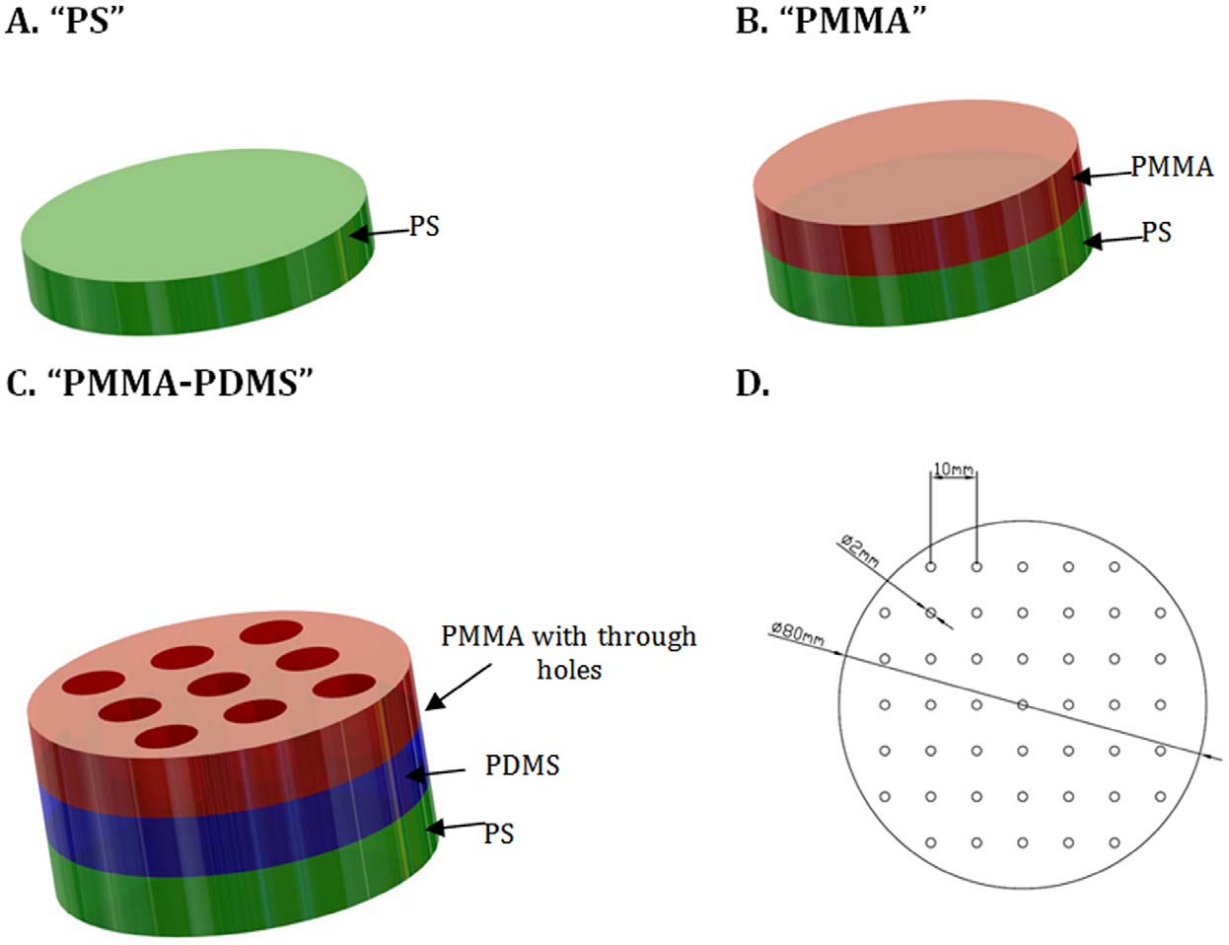
Three different experimental PC12 cells culture conditions for DNA microarray study. (A) laminin-coated PS cell culture dish. (B) laminin-coated 2-mm thick PMMA plate placed inside the cell culture dish. (C) laminin-coated 2-mm thick PMMA plate with through holes placed on the 2-mm thick PDMS layer. (D) A schematic view of a 2 mm thick PMMA plate with through holes placed on the PDMS substrate.

**Figure 2:**
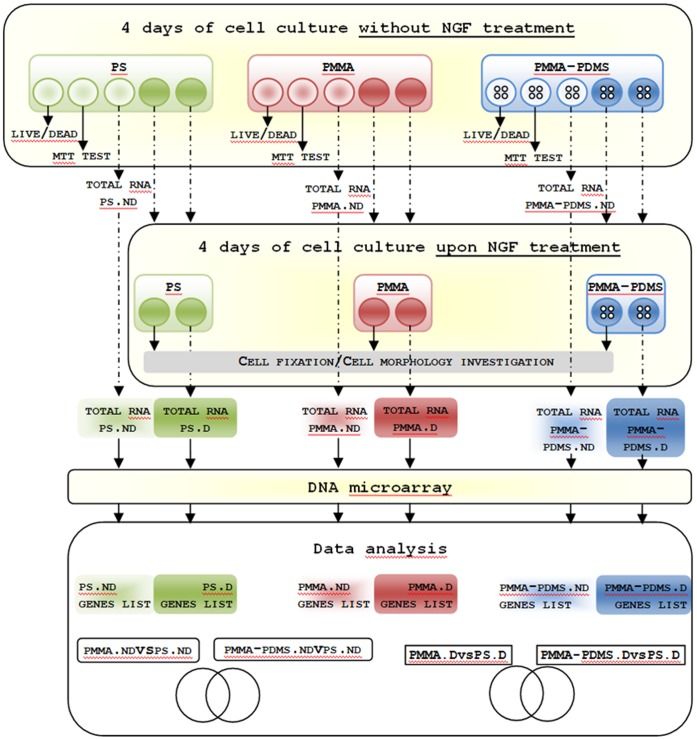
The main experimental and analytical steps of the reported study. The growth and differentiation of PC12 cells were investigated on 2 different polymeric materials: polystyrene (PS) and poly(methyl methacrylate) (PMMA). To determine trans acting effect of PDMS, differentiation was also initiated on PC12 cells seeded on perforated PMMA resting on a PDMS layer. In the next step, we examined whether the cell morphology changes between different polymeric materials can be identified (Fig. 4). Flow cytometry was employed to analyze cell cycle phases in all tested conditions (Fig. 5). For comprehensive examination of the possible effects of three different polymeric materials, DNA microarray based on transcription profiling was employed. PS.ND denotes cells growing on polystyrene, PS.D denotes cells differentiating on polystyrene, PMMA.ND denotes cells growing on PMMA, PMMA.D denotes cells differentiating on PMMA, PMMA-PDMS.ND denotes cells growing on PMMA resting on a PDMS slab and PMMA-PDMS.D denotes cells differentiating on PMMA resting on a slab of PDMS.

The experiments were performed using NimbleGen Rattus norvegicus 12×135K Array that consisted of 26,419 complementary DNA (cDNA) spots. Ten micrograms of the appropriate RNA (PS.ND, PMMA.ND, PMMA-PDMS.ND, PS.D, PMMA.D, PMMA-PDMS.D, see Fig. 1 and Fig. 2 for definitions) were processed and labeled using the standard NimbleGen protocol. Briefly, RNA was converted into cDNA using the SuperScript II cDNA Conversion Kit (Invitrogen, USA). cDNA was random-primed labeled with Cy3-nonamers and hybridized to the microarrays for 16 hours at 42°C. The arrays were washed, dried, and scanned at 5 µm resolution using a GenePix 4000B microarray scanner (Molecular Devices, USA). Data were extracted from scanned images using NimbleScan software (Roche NimbleGen, USA).

### Data Analysis using R and Bioconductor

Data analysis was performed using the Bioconductor packages and the statistical program R (version 2.10.1), as described in [34]. The limma package was used to determine differentially expressed genes between the experimental groups, by fitting a linear model to the expression data for each gene [35], [36]. The data was normalized using the RMA algorithm offered by the oligo package, consisting of three preprocessing steps: convolution background correction, quantile normalization, and a summarization via median polish to the raw data of the obtained expression arrays [37], [38]. To assess the differentially expressed genes, the Empirical Bayes method implemented in the limma package was used. The resulting test statistics is a *moderated t-statistics,* which operates on a weighted average of the single-gene estimated variances *s_g_^2^* and a global variance estimator *s_0_^2^* instead of only *s_g_^2^* [35]. Multiple hypotheses testing were controlled by applying the false discovery rate (FDR) algorithm. For the limma package to assess differentially expressed genes from the microarray data, two matrices need to be specified: the design matrix and the contrast matrix [36]. The design matrix contains the information about the different RNA samples, which were hybridized to the microarrays: PS.D, PMMA.D, PDMS.D, PS.ND, PMMA.ND, PMMA-PDMS.ND. The contrast matrix combines the coefficients designated by the design matrix into contrasts of interest. For example, for non-differentiated PC12 cells following contrasts were investigated: PMMA.ND versus PS.ND (PMMA.NDvsPS.ND), and PMMA-PDMS.ND versus PS.ND (PMMA-PDMS.NDvsPS.ND). In the same way, expression of the genes of interest was assessed for NGF-differentiated PC12 cells. The genes of interest were selected as those displaying an expression difference more than two-fold between the compared cell culture conditions. A Venn diagram was utilized, representing each contrast as a circle enclosing the number of more than two fold regulated genes (up and down). The number of genes similarly regulated on more than one contrast was presented in the overlapping region of the corresponding circles. The top20 most highly differentially expressed genes, between NGF-differentiated and non-differentiated PC12 cells, were revealed for each cell culture condition by using *topTable* function, and the similarly expressed genes at all cell culture conditions were identified.

Functional Gene Ontology (GO) annotation of genes of interest was performed using DAVID Bioinformatics Resources 6.7 [39], [40] to identify functional gene groups and ontology terms that are significantly overrepresented among the genes of interest. To remove any redundancy in our gene list, i.e. when two or more IDs represent same gene, DAVID gene ID was used as unique identifier.

## Results

Growth (no NGF treatment) and differentiation (NGF treatment of cells) of PC12 cells were investigated on both PS and PMMA. To determine the effect of PDMS, PC12 cells were cultured on a perforated PMMA sheet resting on a PDMS layer (Fig. 1). The PMMA sheet was perforated to ensure that possible soluble compounds from PDMS were distributed evenly to the cells. The cell culture models shown in Fig. 1 were chosen in order to be compatible with the requirements of the large number of cells (up to several million) needed for several of the bioanalytical methods used in this study.

The objective of this study was to investigate the biocompatibility of PMMA and PDMS as cell-culturing material for supporting PC12 cell growth and differentiation, as shown in Fig. 2.

### Cell Viability and Metabolic Activity

**Figure 3:**
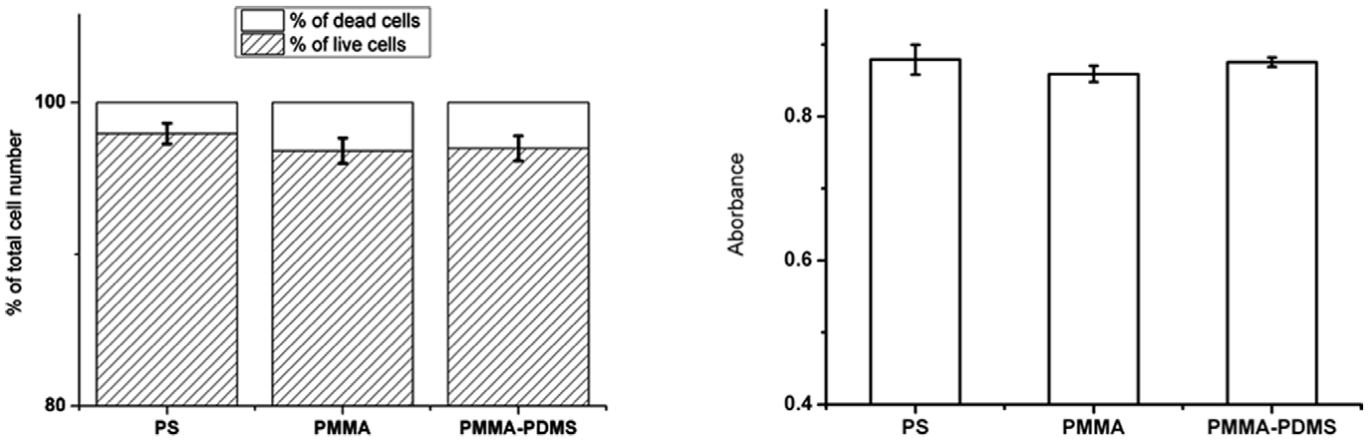
A. Cell viability on the tested samples (Calcein/PI test). B. Measurement of cells metabolic activity in response to the different substrates – MTT test. Error bars represent mean ± SD from three independent experiments.

The cell viability and cells metabolic activity (Fig. 3) of PC12 cells was analyzed after 4 days of cultivation in the absence of NGF. Cells grown on tissue-grade PS was used as the reference condition. As seen, the cell viability and cell death frequencies were comparable at all three cell culture conditions with about 97% of the cells viable and 3% dead in the cultures (Fig. 3A). Cells, seeded with the same density, displayed similar metabolic activity at the three different culture conditions (Fig. 3B), as measured with the MTT test.

### Cell Morphology

**Figure 4:**
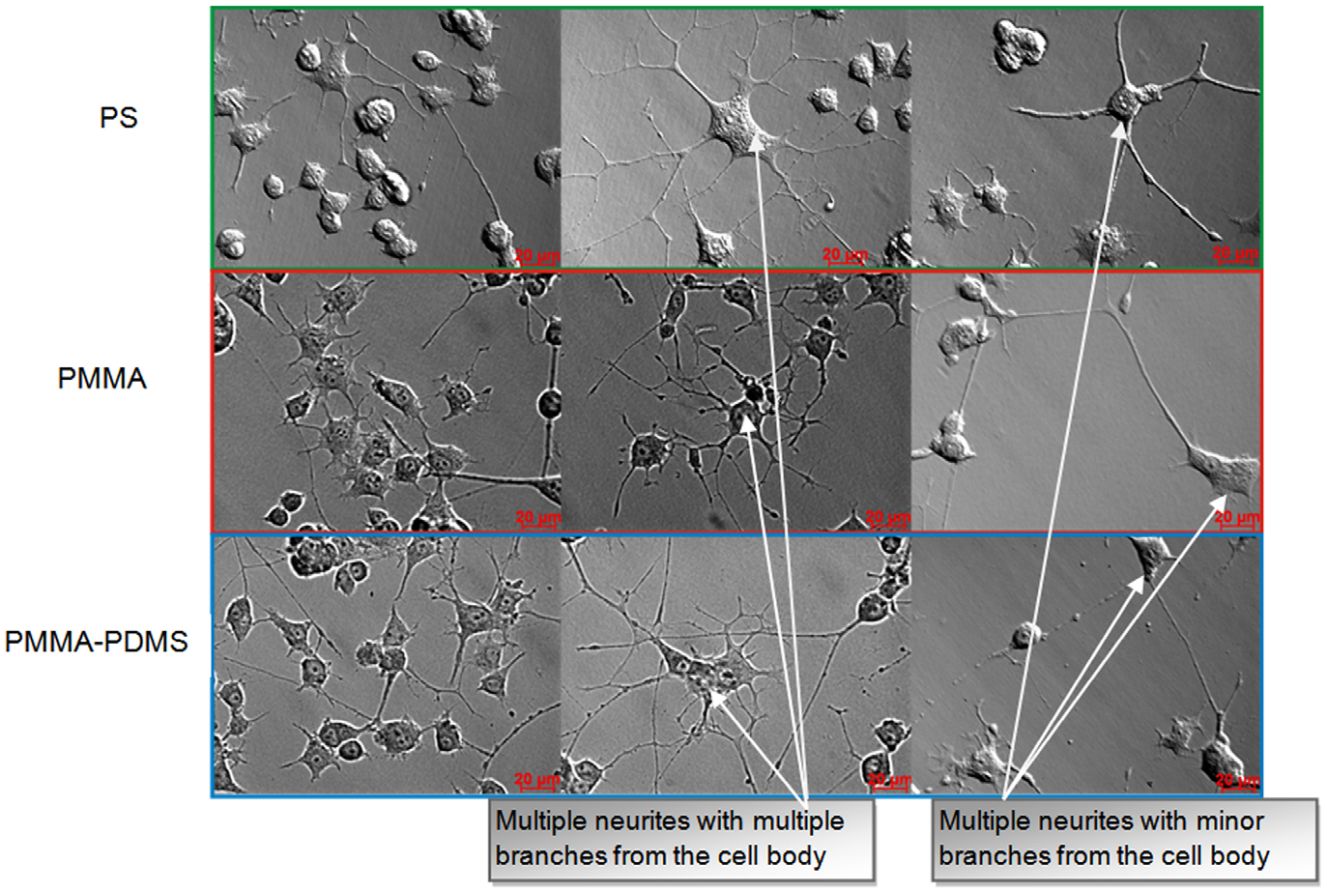
Morphological changes of PC12 cells cultured on: PS (green frame), PMMA (red frame), PMMA-PDMS (blue frame) after 4 days of NGF treatment. Different types of neurites can be found on all polymeric materials used in the presented study: multiple neurites from the cell body with multiple branches, and multiple neurites from the cell body with a minor branch.

In the absence of NGF, the PC12 cells were relatively small and round-shaped (data not shown). Treatment with NGF for 4 days resulted in larger cell bodies and extended neurites of the cells (Fig. 4).

No difference in morphology and neurite network magnitude between PC12 cells was observed when grown and differentiated at the three different cell culture conditions. Multiple neurites from the cell body with multiple branches and multiple neurites from the cell body with a minor branch were seen in all cultures (Fig. 4). The fraction of 4-days-NGF-differentiated cells expressed, as the percentage of total cell number, was similar for the three different culture conditions: PS −51±3%, PMMA −52±2%, PMMA-PDMS −50±2%.

### Cell Cycle Analysis and Sub-G1 DNA Measurement

**Figure 5:**
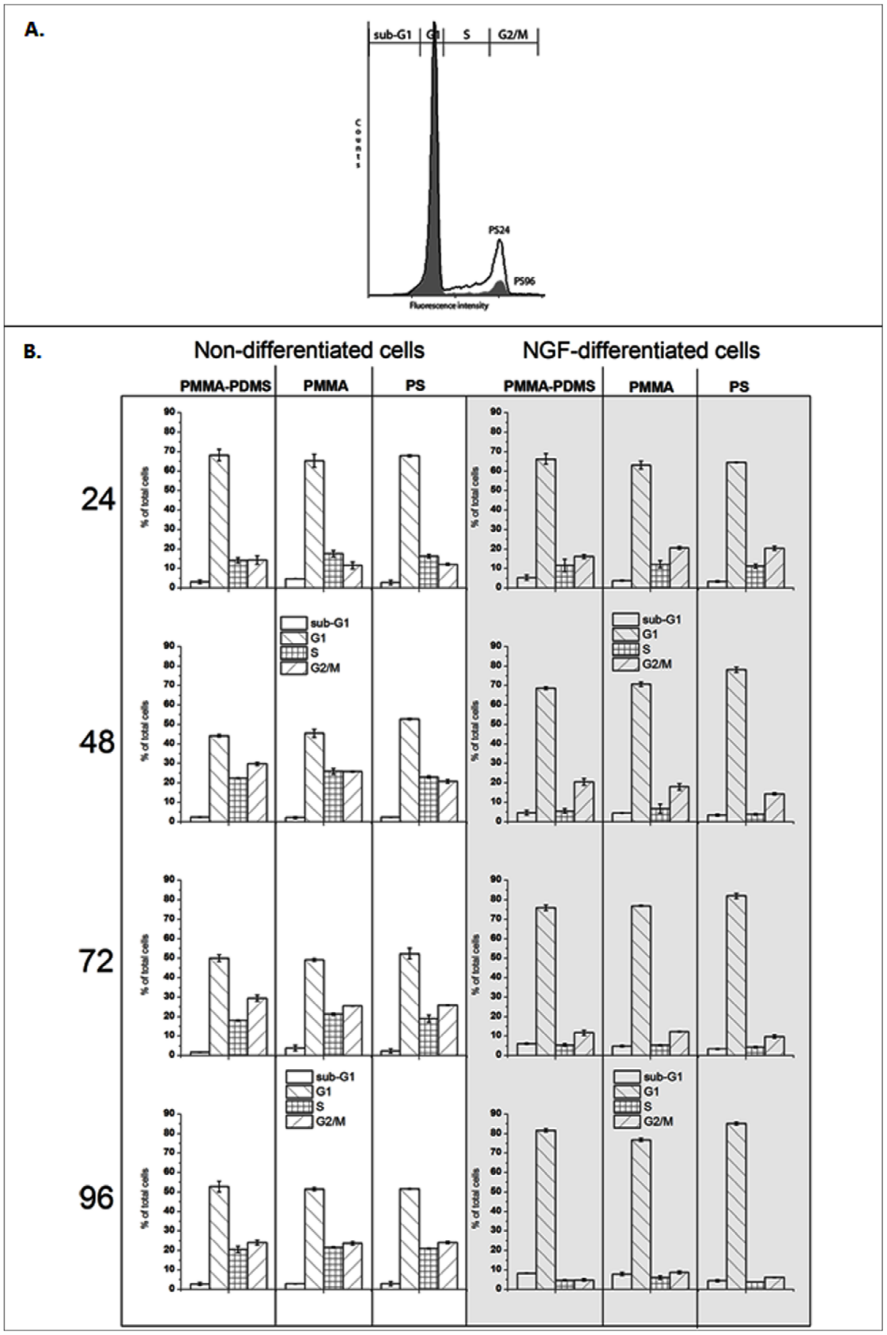
A. The overlay histogram for PC12 cells treated with NGF for 24 hours (blank graph) and for 96 hours (solid, grey graph). The Cyflogic program provided the estimate of percentage of cells in each of the intervals: sub-G1, G1, S, and G2/M. B. PC12 cells were collected after 24, 48, 72, 96 hours from cell seeding (left column, non-differentiated cells), and afterwards 24, 48, 72, 96 hours from NGF treatment (right column, differentiated cells). Samples were analysed by flow cytometry, as described in *Materials and Methods*. Error bars show mean ± SD from three independent experiments.

PC12 cells were collected after 24, 48, 72, and 96 hours, both without and with NGF treatment and subsequently analyzed by flow cytometry (Fig. 5). An example of cell cycle analysis is presented in Fig. 5A: cell cycle results are presented as a histogram and the estimation of percentage of cells in each of the intervals: sub-G1, G1, S, and G2/M was provided by the Cyflogic program. The overlay histogram (Fig. 5B) presents PC12 cells treated with NGF for 24 hours (blank graph) and 96 hrs (solid, grey graph).

In general, for non-differentiated and NGF differentiated cells (Fig. 5), cell cycle phase distribution and number of cells with less DNA content than 2N (sub G1 population) were similar for the three different culture conditions. Although some differences can be observed between non-differentiated cells after 48 and 72 hrs, after 96 hrs the number of cells in various phases was very similar for the three culture conditions. These results are in a good agreement with the results from the cell viability and metabolic activity tests (Fig. 3).

For NGF differentiated PC12 cells, the number of cells in the G1 and sub-G1 phases increased with time while the number of cells in G2/M and S phases decreased. The only exception to this trend was that the amount of cells cultured on PMMA-PDMS for 48 hours in G2/M was higher than in G2/M after 24 hours (Fig. 5). The number of sub-G1 cells after 96 hours of culturing upon NGF treatment was 5% for PS and 8–9% for PMMA and PMMA-PDMS. Overall, the results are consistent with the notion that PC12 cells stop dividing when differentiating into nerve cells as induced by NGF.

### Gene Expression Profiling

For a comprehensive examination of the possible effects of the three different cell culture models, DNA microarray based transcription profiling was also employed. The effect of cell culture condition on (1) non-differentiated cells, (2) the differentiation process and 3) the resulting differentiated neuronal-like cells was of special interest.

**Figure 6:**
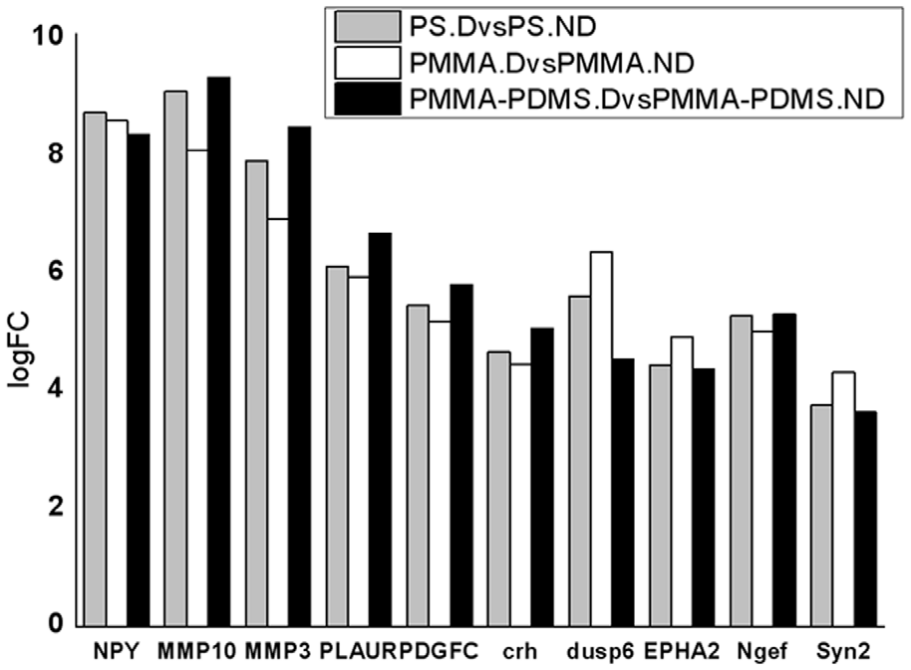
The *top*10 differentially expressed *genes* between NGF-differentiated and non-differentiated PC12 cells similarly expressed in all culture conditions: PS, PMMA, PMMA-PDMS. Notice: the magnitude of the fold-change is similar at all applied culture conditions.

The gene expression profile of non-differentiated cells on PS, PMMA and PMMA-PDMS did not differ significantly (data not shown), indicating that PMMA and PDMS had little impact on non-differentiated cells. The gene expression was significantly different before and after differentiation into neuronal-like cells grown on all three cell culture conditions. This correlates with changes in morphology and inhibition of cell division (Fig. 4 and 5). The top10 similarly regulated genes during differentiation at all cell culture conditions (PS, PMMA, PMMA-PDMS) were associated with neuronal cell development (Fig. 6 and Tab. 1) and correlated well with the observed morphological changes (Fig. 4). This indicated that the resulting neuronal-like cells behaved similarly on PS, PMMA and PMMA-PDMS. To verify this, the gene expression profiles of differentiated nerve cells grown at different cell culture condition were compared.

**Table 1:** Gene ontology processes and pathways associated with the similarly expressed genes at all tested conditions.

*GO Biological Process/Pathway*	*Gene Symbol*
GO:0050877 neurological system process	*NPY, CRH, SYN2*
GO:0007166 cell surface receptor linked signal transduction	*NPY, CRH, PDGFC, EPHA2*
GO:0006508 proteolysis	*MMP10, MMP3*
GO:0042445 hormone metabolic process	*CRH*
GO:0006469 negative regulation of protein kinase activity	*dusp6, Ngef, NPY, PDGFC,*
KEGG Axon guidance	*EPHA2, Ngef*

**Figure 7:**
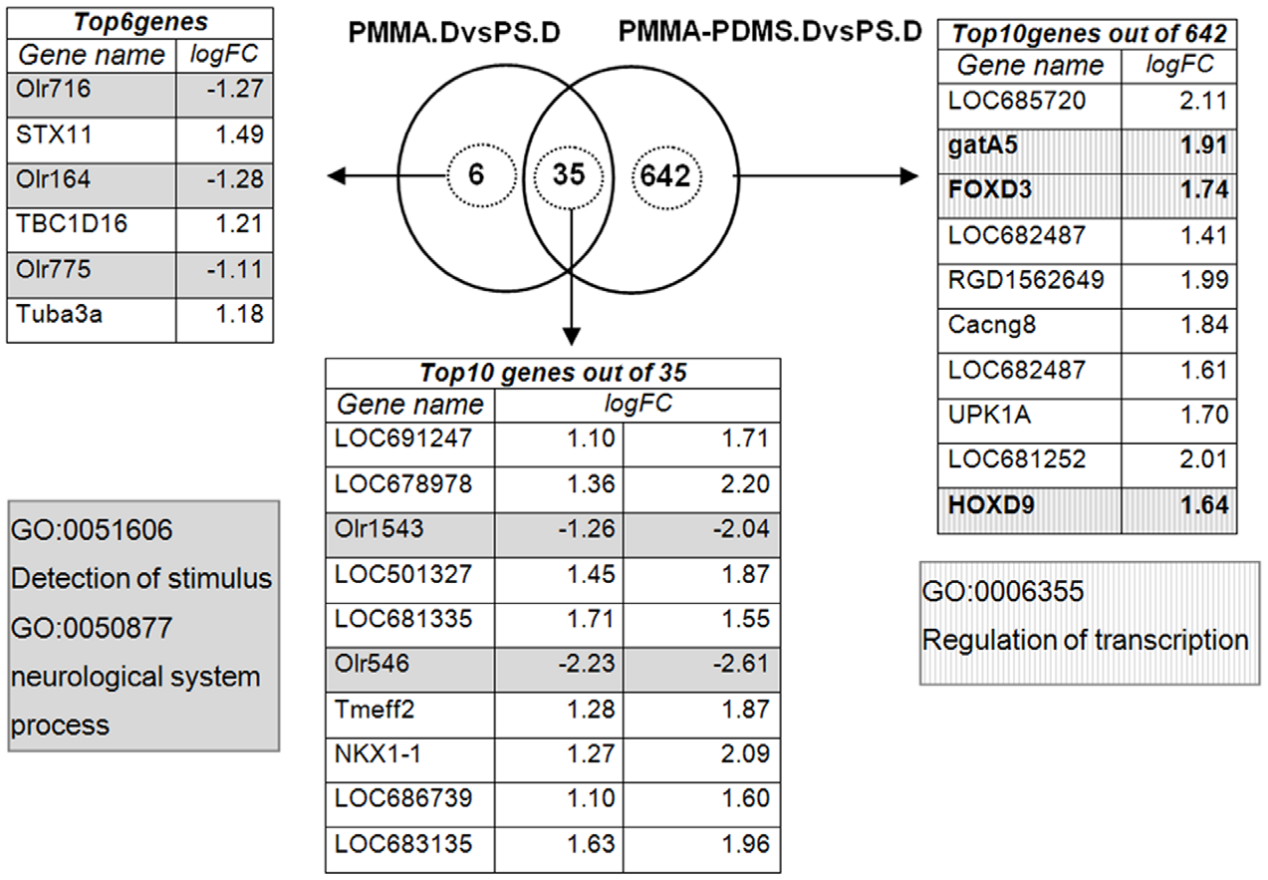
Gene expression profiling of NGF-differentiated PC12 cells. A Venn diagram was utilized representing each contrast as a circle enclosing the number of more than two fold regulated genes (up and down) relative to the PC12 cells cultured on PS. The number of genes similarly regulated on more than one contrast is presented in the overlapping region of the corresponding circles. Up to 10 top genes with their calculated logFC are listed for each contrast.

The result of this analysis showed dramatic differences in gene expression in nerve cells grown at different cell culture conditions (Fig. 7): 6 genes were up regulated and 35 were down regulated in nerve cells grown on PMMA as compared with nerve cells grown on PS. By contrast 642 genes were up regulated and 35 genes were down regulated in nerve cells grown on PMMA-PDMS compared to nerve cells grown on PS. The regulated genes were involved in neuronal cell development and function (Fig. 7 and Tab. 2), indicating that PMMA as well as PDMS in combination with PMMA create conditions that are not the same as PS.

**Table 2:** Functional classification clustering analysis presenting gene ontology processes associated with the 642 differentially expressed genes in NGF-differentiated PC12 cells cultured on PMMA-PDMS versus PS (Enrichment Score>0.5, Final group member threshold – minimum 10 genes).

Term^a^	Count	PValue^c^
**Annotationcluster 1, Enrichment Score^b^: 9.98**		
GO:0007600 sensory perception	71	5.74E-12
GO:0050877 neurological system process	75	1.93E-09
GO:0007166 cell surface receptor linked signal transduction	89	1.06E-07
**Annotationcluster 2, Enrichment Score^b^: 1.65**		
GO:0048562 embryonic organ morphogenesis	10	2.89E-03
**Annotationcluster 3, Enrichment Score^b^: 1.55**		
GO:0045449 regulation of transcription	47	9.10E-03
**Annotationcluster 4, Enrichment Score^b^: 0.84**		
GO:0030182 neuron differentiation	19	5.48E-03

a. Terms based on GOTERM_BP_FAT category, DAVID Bioinformatics Resources 6.7.

b. The overall enrichment score for the group based on the EASE scores of each term members.

c. P-value (or called EASE score) to determine the significance of gene-term enrichment with a modified Fisher’s exact test (EASE score).

## Discussion

Despite being widely used in cell culture chip, only a few reports have investigated the effects of PDMS on cells in greater detail. These reports have furthermore been suggesting that PMDS significantly affect cells by releasing uncured oligomers in the medium [19]. The present study was designed to study PMMA as cell culture substrate and possible factors released from PDMS (Fig. 1). There are abundant examples in the literature showing that PDMS based chips can support cell growth and survival, suggesting that the impact of released oligomers is little. These results are however not necessarily contradictory. PDMS effects might be sufficiently subtle not to affect major cell functions, such as proliferation, apoptosis and cell morphology (Fig. 3,4,5) while still having significant impact on the molecular machinery of cells (Fig. 6). As noted before, cell growth can be unaffected while large changes in gene expression can still be observed [28]. PMMA is an alternative material to produce microfluidics chips in, which sometimes can be biocompatible and sometimes not [26], [28], [41] and as PDMS, the compatibility is apparently dependent on cellular context. We have cultured endothelial cells (HUVEC), epithelial cells (HeLa) [26], [28], fibroblasts, mesenchymal stem cells, embryonic stem cells, adipocyte derived stem cells on PMMA. Corresponding long list of cells grown in PDMS cell culture chips is present in the literature. However, there is lack of information what PDMS (and PMMA) do on the molecular level. Molecular similarities and dissimilarities are context dependent as demonstrated here. There were no molecular differences in cells non-differentiated on PMMA and PS respectively (and also PMMA-PDMS vs. PS), but the corresponding neuronal-like cell cultures showed large differences in gene expression (Fig. 6 and data not shown), which indicate that factors released from PMDS might have little effect on proliferation in general while still having large impact on other cell processes.

The cell cycle analysis revealed that the amount of cells in S phase and G2 phase was decreasing for all tested cell culture conditions during the differentiation process, indicating that more and more cells exit the cell cycle to enter the differentiation process. One exception was however observed. The amount of cells in the phase G2/M for the PMMA-PDMS material after 48-hour of NGF treatment was higher than after 24-hour period (Fig. 5). Even if the G2/M phase is as low at later time points for PMMA-PDMS as the other cell culture condition, it is possible that differentiation on PDMS-PMMA is slower than on PMMA alone and on PS, which might explain the observed differences in gene expression (Fig. 6 and Tab. 2).

PC12 cells represent a well-established *in vitro* model to examine neuronal processes, assessing the viability, growth, proliferation, as well as the differentiation process from a dividing cell into a sympathetic-like neuron cell. According to previous results, maximum induction of PC12 differentiation, upon β-NGF treatment, occurs after 6 to 8 days [42], [43] where 60–100% of PC12 cells in a culture differentiate into neurite protruding cells, indicating a differentiated status [42], [43]. At day 4 after β-NGF induction, approximately 50% of the cells in the culture had neurite protrutions (Fig. 4). Since maximum neurite length is dependent on time [42], [43], it is plausible that some cells have differentiated into an early stage of a nerve cell, but not yet extended any significant neurites. Support for this notion is that the cell cycle analysis showed that the majority of cells had left the cell cycle 4 days after β-NGF induction (Fig. 5), indicating that more cells might be on the way to be differentiated than visualized by the morphological examination (Fig. 4). Overall, the results of the PC12 cell growth, proliferation and differentiation analysis at the three different cell culture conditions seem to be in general agreement (Fig 4) with what has previously been reported, i.e. that NGF promotes neuronal differentiation associated with neurite extensions [44], [45].

Neuropeptide Y (NPY), synapsin II (SYN2), corticotrophin-releasing hormone (CRH), Eph receptor A2 (EPHA2), and neuronal guanine nucleotide exchange factor (Ngef) might be considered as universal markers for PC12 cell differentiation towards neurons by NGF on PS, PMMA and PMMA-PDMS in the tested configurations (Tab. 2). Three genes (NPY, SYN2, CRH) are involved in the neurological system process while EPHA2 and Ngef are engaged in the *Axon guidance* process. This is supported by a number of other studies, indicating that NPY mRNA levels increase during nerve growth factor treatment of PC12 cells [31], [46], [47]. Synapsins were proposed as the optimal candidates to modulate neurite outgrowth in the first stages of neuronal development, owing to their abilities to modulate polymerization and assembly of actin [48]. Recently, it has been demonstrated that corticotrophin-releasing hormone facilitates the outgrowth of axon in spinal neurons [49]. As reported by Sahin and colleagues [50], neuronal guanine nucleotide exchange factor plays an important role in Eph-mediated axon guidance by promoting outgrowth in the absence of ephrins and retraction in the presence of ephrins. Our studies revealed in addition that two types of matrix metallopeptidases (MMPs), MMP3 and MMP10, are involved in NGF-differentiation of PC12 cells on the tested materials (Fig. 6). It has been reported that MMPs are expressed during neuronal development and are up-regulated in nervous system diseases [51]. The reduction in stable focal adhesion points caused by MMPs leads to extracellular matrix degradation and subsequent cell migration [52].

Previously, other gene expression profiling studies have been conducted in PC12 cells [53], [54]. In the first report, longer treatment (9 days) with NGF was applied. To identify NGF regulated genes, a serial analysis of gene expression (SAGE) technology was utilized [53], [54]. These studies and the present study could identify that S100 calcium-binding protein A4 (S100a4) and stathmin-like 4 (stmn 4) genes were regulated during differentiation. In the second study [54], PC12 cells were cultivated for 4 days in DMEM containing 1% horse serum in the presence or absence of 100 ng/ml NGF or 100 ng/ml IGF-1 (insulin-like growth factor-1). Affymetrix Rat RG_U34A_Genechips, which detect mRNAs for approximately 8,800 known genes and ESTs, were employed to assess the difference between NGF (or IGF-1) treated and non-treated cells. Both NGF and IGF-1 are potent trophic factors, which means that they can maintain cell viability as a sole factor, but IGF-1 does not support PC12 cell differentiation. Therefore differentially regulated genes by IGF-1 were removed from a set of NGF-induced genes, and the list of 66 genes with two-fold change between non-treated and treated cells were obtained. Three genes, namely synapsin II, GAP-43, and presenilin-2 were considered as neural specific. Those genes were also found in the present study. It should be noted that tyrosin hydroxylase, which is one of the genes required for production of dopamine was found to be equally expressed in non-differentiated as well as NGF differentiated PC12 cells. This confirms previous findings analysing gene expression [54]. Also undifferentiated PC12 cells have been shown to release dopamine without being treated by NGF [55], [56].

Biocompatibility investigations of microfluidic system can be divided into two approaches with increasing complexity. The first approach is investigating the biocompatibility of a material. Such investigations are usually performed in batch cultures and the reference point is usually a commercial cell culture flask [6]. The second approach is to test the whole microfludic system and compare the results to batch cultures in cell culture flasks. Microfluidic cell culturing can have multiple additional effects on the cells besides effects caused by the material of the system. The surface to volume of a microfludics system is typically large as compared to batch cultures, which increases the effects of the material in relation to adsorption and absorption effects. Furthermore, cell cultures in microfludic chips are usually continuously perfused, resulting in effective removal of waste product from cells, removal of released possibly cytotoxic compounds from the material like PDMS, but also removal of autocrine and paracrine factors essential for the cells. It is therefore very difficult to link effects of a material when investigating effects of complete microfluidics systems. In addition, many of the powerful analysis tools such as flow cytometry and gene expression profiling requires relative large number of cells, which can be difficult to obtain from microfluidic systems.

In conclusion, the results show that a piece of PDMS underneath a perforated PMMA alone can lead to significant variations of gene expression profiles compared to PMMA and PS (Fig. 6). Due to the extensive use of PDMS and its reported negative effects on cells, it is highly important to improve the understanding on how PDMS affect cells. Moreover, detecting additional NGF-responsive genes enables new knowledge how trophic factors perform their functions [53].
